# Effect of Amphotericin B Nanodisks on *Leishmania major* Infected Mice

**DOI:** 10.4172/2153-2435.1000311

**Published:** 2014-09-29

**Authors:** PA Cole, JV Bishop, JA Beckstead, R Titus, RO Ryan

**Affiliations:** 1 Microbiology, Immunology, and Pathology Department, Colorado State University, Fort Collins, CO 80523-1619; 2 Children’s Hospital Oakland Research Institute, 5700 Martin Luther King Jr. Way, Oakland CA 94609, USA

## Abstract

**Objective:**

To assess the efficacy of a novel formulation of the polyene antibiotic, amphotericin B (AMB), as therapy for cutaneous leishmaniasis in different mouse strains.

**Methods:**

(AMB), was formulated into water-soluble transport particles, termed nanodisks (ND). Balb/c and CH_3_ mice infected with *Leishmania major* on Day 0 were administered vehicle alone, empty ND or AMB-ND on Day 1 and day 7, via the tail vein. Mice were sacrificed 25 or 50 days post inoculation and tissue histology evaluated. Balb/c mice treated with vehicle or empty ND showed signs of severe infection while CH_3_ mice had less inflammation and fewer parasites. AMB-ND treatment (2 mg/kg) had a marked therapeutic effect on *L. major* infected Balb/c mice and a discernable therapeutic benefit on CH_3_ mice.

**Conclusions:**

AMB-ND is efficacious in the treatment of cutaneous leishmaniasis in both susceptible and resistant mouse strains. It may be inferred that AMB-ND may be useful for prophylactic and/or treatment of early stage *Leishmania spp.* infection.

## Introduction

Parasites of the genus *Leishmania* are intracellular protozoans that infect more than 12 million individuals worldwide, with 400,000 new cases each year. Widespread in over 80 countries around the globe, leishmaniasis has been identified as an important medical problem for military personnel serving in endemic regions [1]. Occurring in several forms, disease is most often seen as non-fatal cutaneous lesions although epidemics of the potentially fatal visceral form may occur and have caused thousands of deaths. Parasites are transmitted to humans via the bite of sand flies (subfamily *phlebotomina*). These tiny sand-colored, blood-feeding flies breed in forest areas, caves, or the burrows of small rodents. In addition to humans, wild and domesticated animals can serve as a reservoir of infection. Over 20 species and subspecies of *Leishmania* infect humans, each causing a spectrum of symptoms. These range from simple, self-healing skin ulcers (e.g. infection with *L. major*) to severe, life-threatening visceral disease (e.g. *L. donovani or L. chagasi*). Common symptoms include fever, malaise, weight loss and anemia as well as splenomegaly and hepatomegaly in the visceral form.

In recent times, leishmaniasis has emerged as an opportunistic infection in HIV positive individuals [2,3]. Visceral leishmaniasis in individuals co-infected with HIV has been reported in both endemic and non-endemic areas. Most at risk for infection are HIV-positive intravenous drug users with a low CD4 T cell count and high HIV viral load. No vaccines exist for visceral leishmaniasis and conventional chemotherapy is inadequate. A common therapeutic regimen involves intravenous administration of pentavalent antimonials (meglumine antimoniate and sodium stibogluconate) as primary therapy. During the past decade, large-scale failure (up to 64%) of pentavalent antimonial agents in patients from India prompted its abandonment as first-line treatment. An alternative drug is the membrane-active alkylphospholipid, hexadecylphosphocholine (Miltefosine), which inhibits enzymes of ether lipid metabolism [4]. This drug is effective because *Leishmania* spp. possess high levels of ether lipids, mainly in the phospholipid component of glycosylphosphatidylinositol-anchored glycoproteins present on the parasite surface.

Another drug commonly used in treatment of leishmaniasis is the macrolide polyene antibiotic, amphotericin B (AMB). This hydrophobic, water insoluble molecule is highly active but has toxicity complications [5]. AMB has an affinity for 24-substituted sterols, including ergosterol, which is abundant in fungal and *Leishmania* cell membranes [6]. Different hypotheses have been presented to explain AMB mechanism of action including sterol extraction from membranes [7] and oxidation-dependent processes [8,9]. Another hypothesis [10] proposes that AMB forms membrane pores that cause leakage of cellular contents. The resulting disruption of osmotic integrity leads to cell death. Specificity is achieved by the fact that host membranes contain cholesterol in lieu of ergosterol while toxicity occurs because AMB is also able to bind cholesterol, albeit with lower affinity [11]. Whereas AMB solubilized in deoxycholate micelles is generally toxic, this problem is significantly reduced upon formulation of AMB into liposomes, thereby extending its plasma residence time and improving its pharmacokinetic profile [12].

A novel lipid formulation of AMB, termed AMB-nanodisks (ND) has been reported [13]. AMB-ND exist as water soluble nanoscale assemblies of phospholipid, apolipoprotein scaffold and AMB [14]. Characterization studies have provided insight into the structural organization of these complexes [15] and *in vivo* studies in mice have revealed potent biological activity. In studies of *L. major* infected Balb/c mice, AMB-ND showed greater efficacy than liposomal AMB following intraperitoneal administration [16]. Recognizing that AMB can be toxic, with infusion related reactions and/or hepatic toxicity limiting therapeutic application of this drug, [17] the efficacy and toxicity of intravenously administered AMB-ND in mouse strains that are susceptible and resistant to *L. major*, respectively, has been examined.

## Materials and Methods

### Nanodisks

AMB-ND were formulated essentially as described previously [13]. Briefly, 10 mg of dispersed phospholipid, comprised of 7 mg dimyristoylphosphatidylcholine and 3 mg dimyristoylphosphatidylglycerol were combined with 2.5 mg AMB (USP grade; Research Organics Inc.) and incubated with 4 mg recombinant human apolipoprotein A–I at 24°C. Following sample clearance by bath sonication and dialysis, ND associated AMB was determined spectrophotometrically at 416 nm by dissolving an aliquot of the ND solution in dimethylsulfoxide (extinction coefficient = 1.214 × 10^5^M^−1^cm^−1^). Sterile filtered (0.22 m) AMB-NDs were stored in the dark at 4°C for < 40 days. Empty ND lacking AMB were formulated in the same manner. ND preparations were diluted in phosphate buffered saline (PBS) to the respective concentrations for each treatment group.

### Parasites

*Leishmania major* parasites from the LV39 strain were grown on sheep blood agar in parasite growth medium [RPMI-1640 (Sigma-Aldrich, St. Louis, MO) with 5% fetal bovine serum (Hyclone, Logan, UT), 10 mM Hepes (Sigma-Aldrich), 100 U/ml penicillin (Gibco, Carlsbad, CA), 100 mg/ml streptomycin (Gibco), 2 mM L-glutamine (Gibco), 1 mM sodium pyruvate (Gibco), 0.2 mM L-asparagine (Calbiochem, San Diego, CA), 0.6 mM L-arginine (Calbiochem), and 2% sterile-filtered normal human urine]. Parasites were passaged through mice every 2 months to retain virulence and from flask to flask twice a week. At 5– 6 days after flask inoculation, parasites were removed from the growth medium, centrifuged to remove dead parasites, washed in DMEM and counted on a Neubauer hematocytometer prior to re-suspending in DMEM.

### Mice

Eighteen Balb/c mice (susceptible strain) and 18 CH_3_ mice (resistant strain) were employed. Balb/c mice are known as a strain that is susceptible to *L. major* infection and CH_3_ mice are considered resistant to infection. Adult mice (6–10 weeks old) were procured from the National Cancer Institute and housed in colonies at Colorado State University (CSU) under supervision of the CSU Laboratory Animal Resources department with authorization by the Institutional Animal Care and Use Committee. Mice were observed daily throughout the experiment. Mice were anesthetized with Ketamine and Xylazine prior to *L. major* in oculations and treatments. All mice were inoculated with l×10^6^ L. *major* parasites in 50 µL DMEM administered subcutaneously in the left hind footpad. There were 3 treatment groups of 6 mice for both strains. Six mice from each strain were treated with AMB-ND and in order to evaluate potential therapeutic effectiveness of AMB-ND, control mice were injected with an equivalent amount of empty ND lacking AMB or an equivalent volume of PBS. These treatments were administered intravenously via the tail vein at day 1 and day 7 after *L. major* inoculation. The AMB-ND treatment group received 2 mg/kg AMB in 200 µL total volume/mouse. Size progression of footpad lesions was measured in mm’s twice weekly using a Vernier caliper and for each measurement the difference between the infected left foot and the uninfected right foot was determined. Half of the mice of each strain from each treatment group were euthanized in a CO_2_ chamber at day 25 and the other half at day 50 post inoculation.

### Postmortem examination

Gross necropsies were performed. Tissues collected for histologic examination included the infected left foot, non-infected right foot, left popliteal lymph node, liver, spleen, and kidney. These were fixed in 10% neutral buffered formalin. The feet were sagitally sectioned and decalcified in 8% formic acid for 5 days before processing. Tissues were processed routinely, sectioned at 5 µm, and stained with hematoxylin and eosin. Foot sections were evaluated for epidermal ulceration, inflammation and the number of parasites present. Livers were evaluated for inflammation and parasite number while spleens and the popliteal lymph nodes were examined for the degree of lymphoid hyperplasia, the amount of inflammation and the number of parasites. The kidneys were examined for any type of histologic abnormalities.

## Results

### Effect of PBS or empty ND treatment on *L. major* infected Balb/c mice

**Figure 1:**
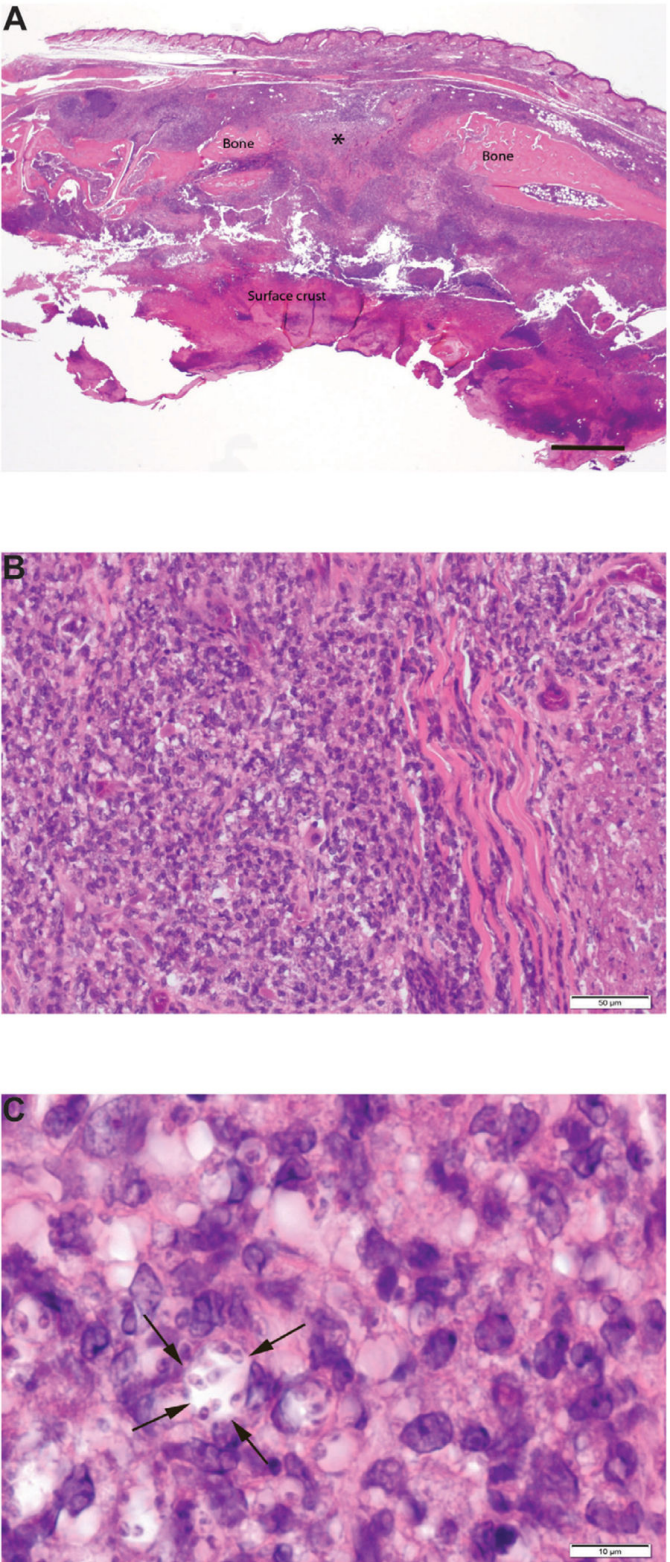
Photomicrographs of a sagittally sectioned foot of a representative Balb/c mouse sacrificed 25 days PI with *L. major* and treatment with PBS. Panel A) The footpad epithelium is ulcerated and the surface has a thick crust of cellular debris, inflammatory cells and fibrin. The subcutaneous tissue has severe chronic inflammation. The central area of the metatarsal bone (*) has undergone lysis due to osteomyelitis. H&E, bar=500µm. Panel B) The subcutis has solid sheets of densely packed mixed mononuclear inflammatory cells. H&E, bar=50µm. Panel C) Note that macrophages have *Leishmania sp.* amastigotes in the cytoplasm. The cell near the center of the photograph has 8 organisms in its cytoplasm (arrows). H&E, bar=10 µm.

Over the time course of the experiment, the infected left foot of both treatment groups became progressively thicker than the uninfected rightfoot, with severe thickening present by day 50. Extensive ulceration of the footpad was grossly evident by day 25. Histologic examination of infected foot tissue collected at day 25 revealed epidermal ulceration, severe pyogranulomatous inflammation in the soft tissues, and osteomyelitis of metatarsal bones. A photomicrograph of the sagittal section of a typical infected foot (Figure 1A and 1B) illustrates a large epidermal ulcer with a thick surface crust of cellular debris, inflammatory cells, and fibrin. In addition, the subcutaneous tissue has extensive inflammation with neutrophils, macrophages, lymphocytes and plasma cells present. The central area (*) of the metatarsal bone has undergone lysis. The haired skin of the dorsum of the foot is intact. Large numbers of *L. major* were present within macrophages located in the soft tissues. Higher magnification of the subcutis of this sagittal section of the foot reveals sheets of closely packed mixed mononuclear inflammatory cells with *L. major* amastigotes present in many macrophages (Figure 1C). The extent of tissue damage at day 50 (not shown) was similar to that at day 25 in these mice, whether treated with PBS or empty ND. The left popliteal lymph nodes were severely reactive at days 25 and 50 in both treatment groups. The number of *L. major* present in the left popliteal lymph nodes varied among mice in the PBS treatment group and was highest in the empty ND treatment group. Nodes with high numbers of parasites also had high numbers of neutrophils. Furthermore, spleens of infected Balb/c mice treated with PBS or empty ND had moderately reactive lymphoid follicles and moderate numbers of neutrophils in red pulp at day 25 and milder changes of a similar nature at day 50. Livers had mild, moderate or severe randomly located accumulations of mixed mononuclear inflammatory cells. No differences in liver inflammation were detected between these treatment groups or between day 25 and day 50.

### Effect of PBS or empty ND on *L. major* infected CH_3_ mice

**Figure 2:**
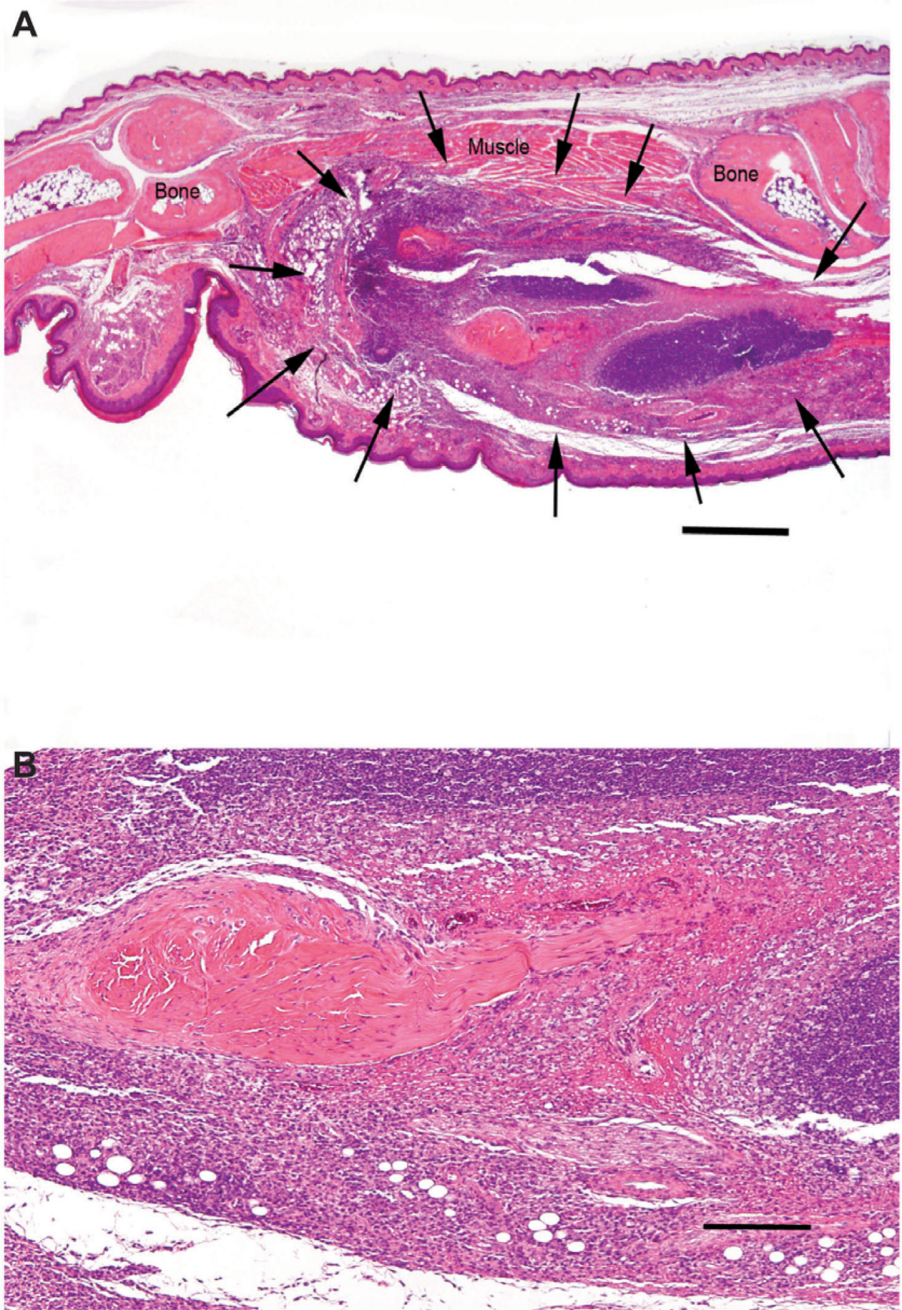
Photomicrographs of a sagittally sectioned foot of a representative CH_3_ mouse sacrificed 25 days PI with *L. mayor* and treatment with PBS. Panel A) Moderate chronic inflammation is present in the soft tissues (arrows). The footpad epidermis and haired skin are intact. The inflammation has not spread to the bones. H&E, bar = 500 µm. Panel B) The subcutis has sheets of closely packed mixed mononuclear inflammatory cells with darkly stained lymphocytes and plasma cells in the upper half of the photomicrograph and more lightly stained macrophages in the lower half. These surround a central area of dense collagenous connective tissue. H&E, bar = 50 µm.

Footpads of CH_3_ mice infected with *L. major* were moderately thickened at day 25 but not thickened at day 50. No footpad ulceration was observed, either on day 25 or day 50 PI. However, histologic examination of sagittal sections of infected feet revealed large numbers of *L. Major* parasites and moderate to severe inflammation in the soft tissues of the infected foot at day 25 (Figure 2A and 2B). By day 50 only a few organisms were present in mice treated with empty ND and none were detected in the PBS treatment group. At 50 days PI, mild inflammation was present in the soft tissues of the infected foot of mice in both treatment groups. The left popliteal lymph nodes of PBS treated mice had mildly reactively mphoid elements and mild neutrophil mild to days 25 and 50. Lymph nodes of mice treated with empty ND had mild to moderately reactive lymphoid elements and mild neutrophil infiltration at day 25, with moderate to severely reactive lymphoid elements and greater neutrophil infiltration at day 50. No *L. major* parasites were found in lymph nodes of infected CH_3_ mice. Likewise, spleens of infected CH_3_ mice treated with PBS or empty ND were unremarkable. Livers had mild inflammation at day 25 but none at day 50. CH_3_ mice had decreased numbers of organisms at day 50 compared to day 25 while Balb/c mice did not show this decrease. Furthermore, CH_3_ mice had less inflammation at days 25 and 50 than Balb/c mice.

### The effect of AMB-ND on *L. major* infected Balb/c mice

**Figure 3:**
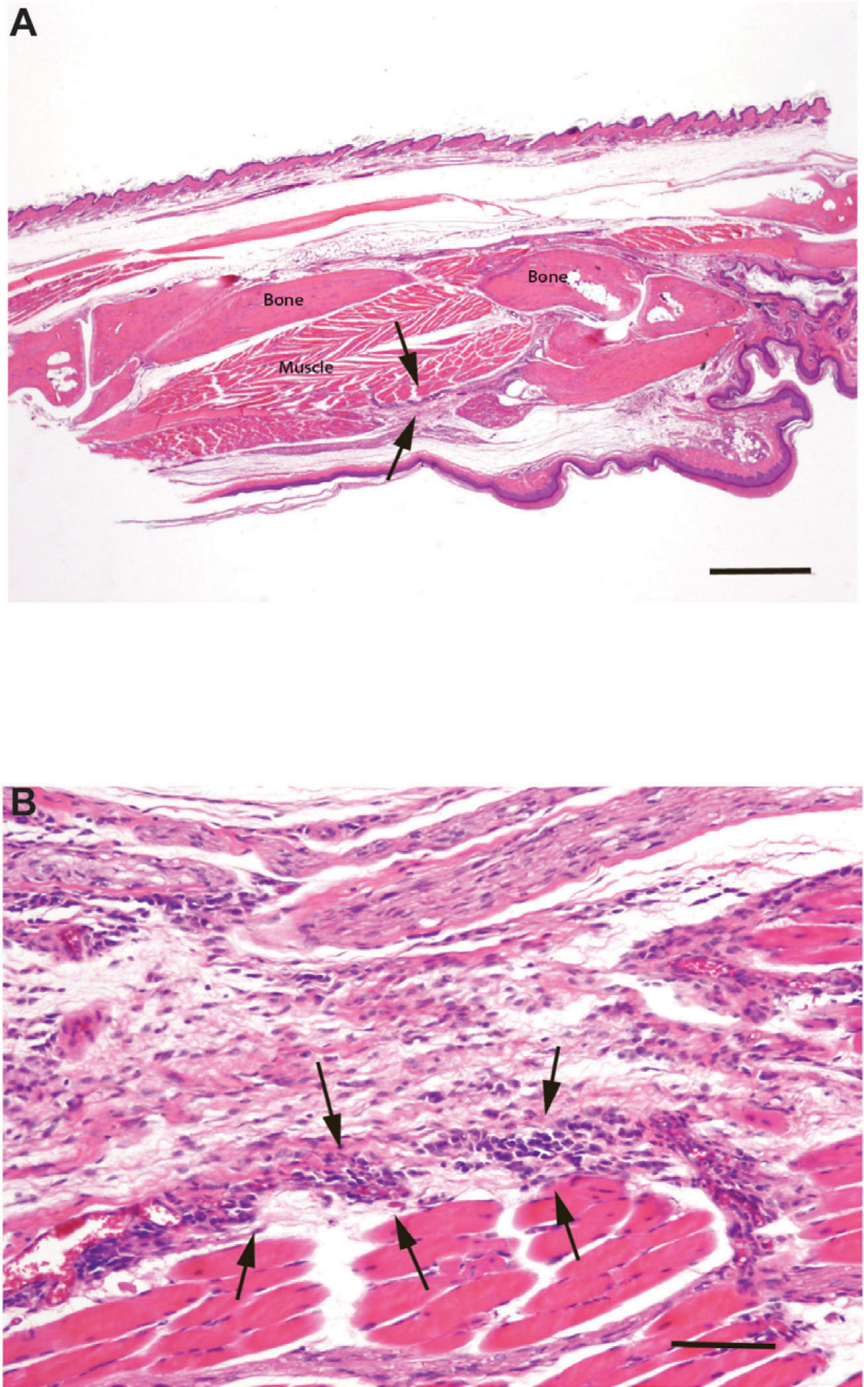
Photomicrographs of a sagittally sectioned foot of a representative Balb/c mouse sacrificed 25 days following inoculation with *L. major* and treatment with AMB-ND. Panel A) Note the mild inflammation in subcutaneous tissue (arrows). The footpad epidermis (bottom) is intact and bone and muscle have no lesions. H&E, bar=500µm. Panel B). The mild focal inflammatory cell infiltrate consists of mixed mononuclear cells (arrows). Normal skeletal muscle lies beneath these cells with normal loose connective above. H&E, bar=500 µm.

In contrast to the PBS or empty ND treatment groups, AMB-ND treated mice manifested minimal change in footpad thickness throughout the experimental period. A small focus of footpad ulceration was present in 1 of 3 mice at day 25, with no ulceration in the other 2 mice. There was no ulceration in 3/3 mice at day 50. Histologic examination revealed that soft tissues of the feet had low numbers of *L. major* parasites and mild inflammation at day 25 PI. Inflammation became moderate by day 50. A photomicrograph of a sagittal section of a typical infected footpad from day 25 illustrates the mild nature of the inflammation (Figures 3A and 3B). Popliteal lymph nodes were moderately reactive and had low numbers of parasites, with mild neutrophil infiltration observed at days 25 and 50 PI. Livers of these mice had little or no inflammation at either day 25 or day 50 PI. Spleens and kidneys had no histologic lesions.

### Effect of AMB-ND on *L. major* infected CH_3_ mice

**Figure 4:**
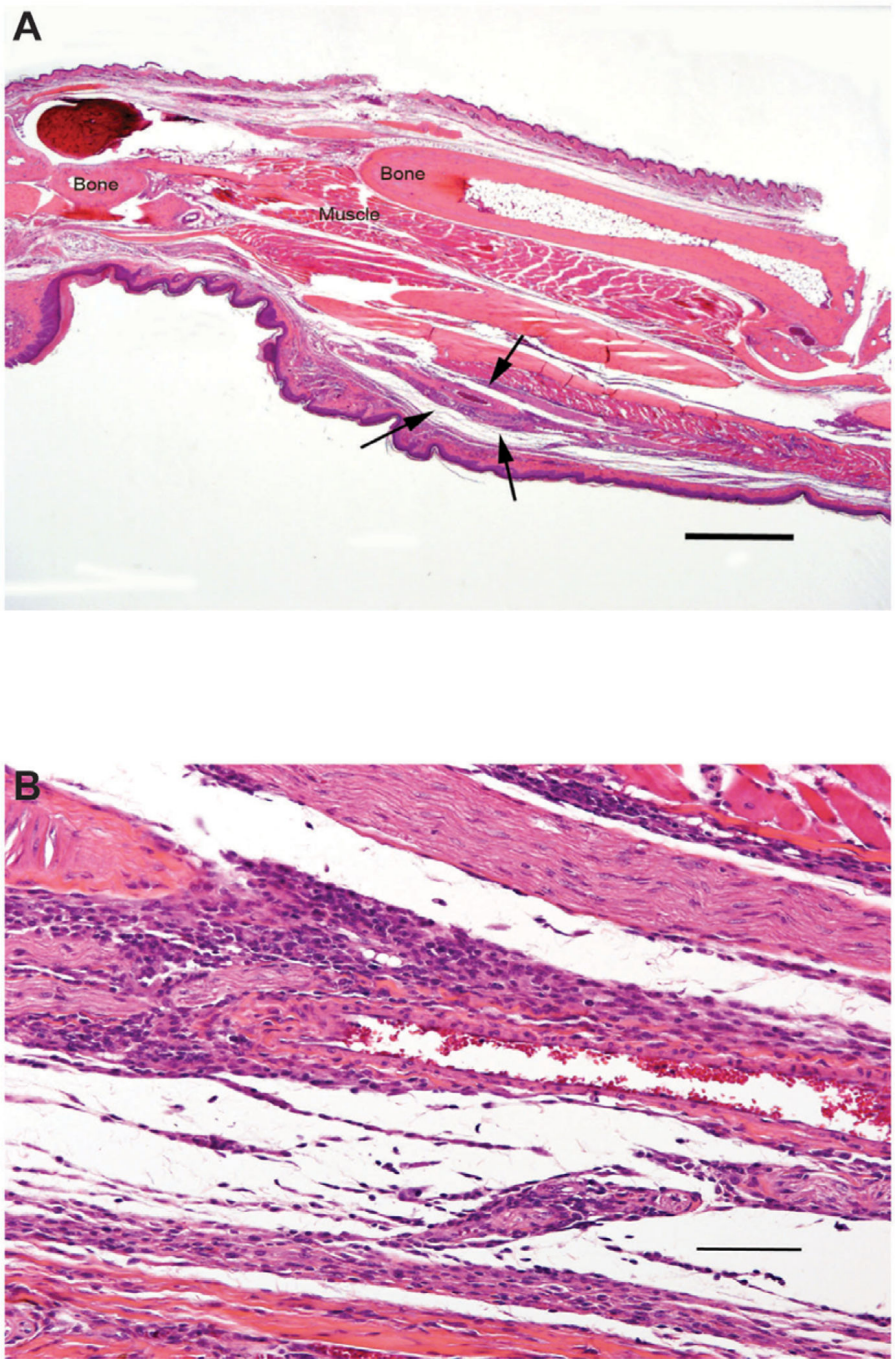
Photomicrographs of a sagittally sectioned foot of a representative CH_3_ mouse sacrificed 25 days following inoculation with *L. major* and treatment with AMB-ND. Panel A) Mild focal inflammation (arrows) is present in the subcutis. H&E, bar=500 µm. Panel B). The cellular infiltrate is composed of mixed mononuclear inflammatory cells. H&E, bar=500 µm.

These mice had no increase in thickness of infected feet and no footpad ulceration. Histologic examination at days 25 and 50 PI showed no detectable *L. major* parasites and mild inflammation (Figures 4A and 4B). Furthermore, there was no change in popliteal lymph nodes, spleens, kidneys, livers, or non-infected right feet.

## Discussion

The gross footpad lesions and histologic findings of organisms and inflammation in the feet of Balb/c and CH_3_ mice inoculated with *L major* and treated with PBS or empty ND indicate that the inoculation regimen employed results in significant infection with accompanying inflammatory lesions. In mice treated with empty ND or PBS, the lesions in Balb/c mice were more severe than those in CH_3_ mice. This indicates that, in general, CH_3_mice are far more capable of resisting an *L. major* infection than Balb/c mice, reinforcing the concept of significant strain differences in response to *L. major* infection [18]. Grossly, CH_3_ mice developed no footpad ulcerations and only slight thickening of the feet. However, histologic examination of the feet showed that organisms and inflammation were present. Although CH_3_ mice were infected, their inflammatory response was less intense than that of Balb/c mice. The genetic basis for this marked difference in susceptibility remains to be deciphered.

It is clear that, in both mouse strains, treatment of *L major* infected mice with AMB-ND reduced the severity of footpad lesions and numbers of parasites remaining in the feet and popliteal lymph nodes. Treatment with empty ND had no discernible therapeutic effect compared to PBS. Minor differences between these two treatment groups were noted but the trends observed in both were similar. The therapeutic effects of AMB-ND were more pronounced in the susceptible Balb/c strain than the resistant CH_3_ strain.

The observation that no histologic lesions were detectable in the kidneys indicates AMB-ND’s are not renal toxic at this dosage, route of administration, and treatment regimen. Furthermore, no other evidence of toxicity, illness or infusion-related effects was detected in mice treated with AMB-ND. Considering that AMB-ND self assemble upon incubation of phospholipid, apolipoprotein and AMB [14], generating product particles that are homogeneous, stable and fully biocompatible, their potential utility as a transport/delivery vehicle is evident. The high AMB binding capacity of ND [15], the potential for lyophilization/rehydration and lower toxicity/enhanced efficacy versus liposomal or deoxycholate formulations of this antibiotic, suggest further study is warranted.

It is remarkable that, even though AMB-ND treatment was confined to day 1 and day 7 PI, therapeutic benefit extended to at least day 50. The prolonged and enduring therapeutic effect seen with AMB-ND treatment suggests prophylactic administration should be considered for individuals planning to spend time in endemic regions. Even with the mild infection seen in CH_3_ mice, AMB-ND treatment was beneficial. Since AMB-ND cleared the infection and reduced inflammation in both strains, it is likely that AMB-ND treatment would be useful for subclinical or early stages of *L. major* infection.
